# Public Awareness of Disability Support Services and Support for Disability-Inclusive Measures in Saudi Arabia: A Cross-Sectional Online Survey

**DOI:** 10.3390/healthcare14213666

**Published:** 2026-10-28

**Authors:** Sahar A. Abduljawad, Ohood K. Almuzaini, Nouf M. Alourfi, Ghadeer A. Joudah, Nasser M. Alorfi, Saad M. Wali

**Affiliations:** 1 Department of Health Administration and Medical Information, College of Al-Leith Health Sciences, Umm Al-Qura University, Makkah 21961, Saudi Arabia; 2 Pharmacology and Toxicology Department, College of Pharmacy, Umm Al-Qura University, Makkah 24381, Saudi Arabia; 3 King Salman Center for Disability Research, Riyadh 11614, Saudi Arabia; 4 Department of Chemistry, Arts and Sciences College, Rabigh Campus, King Abdulaziz University, Jeddah 21589, Saudi Arabia; 5 Department of Pharmacology and Toxicology, Faculty of Pharmacy, King Abdulaziz University, Jeddah 21589, Saudi Arabia

**Keywords:** disability, disability support services, public awareness, support for disability-inclusive measures, disability inclusion, autism spectrum disorder, Saudi Arabia

## Abstract

Background/Objectives: Saudi Arabia has expanded disability support services and rights-based policies under Vision 2030. However, the effectiveness of these initiatives may depend partly on public awareness of available services and societal support for disability-inclusive measures. This study assessed public awareness of disability support services, support for disability-inclusive measures, and factors associated with both domains among adults in Saudi Arabia. Methods: A cross-sectional online survey was conducted across all five regions of Saudi Arabia using a structured self-administered questionnaire distributed through social media with snowball dissemination. A total of 948 adults participated. Exploratory and confirmatory factor analysis identified composite scores for awareness (two items; Cronbach’s alpha = 0.846) and support for disability-inclusive measures (four items; Cronbach’s alpha = 0.845) using five-point Likert-scale responses. A structural equation model was fitted to examine factors associated with both outcomes, with support regressed on awareness to examine their association. Results: The median awareness score was 8.0 (IQR 6.0–9.0) of 10, and the median support score was 19.0 (IQR 17.0–20.0) of 20. More than 84% of participants agreed or strongly agreed with each support item. Awareness was positively associated with support for disability-inclusive measures. Awareness was lower among participants in the Eastern and Northern regions compared with the Central region, and among those with a bachelor’s or postgraduate degree compared with primary-school education. Support for disability-inclusive measures was higher among participants in the Western region and those with secondary-school or bachelor’s-degree education, and lower among those with no or uncertain previous personal contact with a person with a disability, and among those most familiar with hearing impairment. Public awareness campaigns were the most frequently endorsed strategy for improving disability inclusion (75.4%). Conclusions: Public support for disability-inclusive measures in Saudi Arabia was high, while self-reported awareness of disability support services varied across population groups. These findings are associative and, given the cross-sectional design, cannot establish the direction of the relationship between awareness and support. The observed regional and educational differences nonetheless highlight the potential value of targeted public education and communication strategies to strengthen disability inclusion.

## 1. Introduction

An estimated 1.3 billion people, approximately 16% of the global population, live with a significant disability, and this number is expected to increase with population ageing and the growing prevalence of noncommunicable diseases [1]. Persons with disabilities experience persistent inequalities in health, education, employment, and social participation, many of which arise from environmental, institutional, and attitudinal barriers rather than impairment alone [1,2]. The United Nations Convention on the Rights of Persons with Disabilities requires States Parties to promote awareness of the rights, dignity, and capabilities of persons with disabilities and to combat stereotypes and prejudice [3]. Similarly, the 2030 Agenda for Sustainable Development commits countries to ensuring that development is inclusive and that no one is left behind, including people with disabilities [4]. Within this framework, public awareness and societal support are important components of effective disability inclusion, as the availability of rights, services, and supportive policies does not necessarily ensure their recognition, utilization, or broader societal legitimacy. The evidence for this proposition comes largely from work on intellectual disability, where public awareness, attitudes, and beliefs have been studied most systematically [5,6].

Two constructs are examined here, and each is defined at the outset. Awareness refers throughout to respondents’ self-reported familiarity with the existence of disability support services, not to objectively tested knowledge of what those services provide or how they are accessed; the distinction matters because the two need not coincide. Support for disability-inclusive measures refers to endorsement of the principle that specific provisions—priority in healthcare, educational and employment support, accommodations, assistance to families, and public investment in services—are warranted; it is not an assessment of any named policy instrument. Awareness and support so defined represent related but distinct dimensions of disability inclusion. Limited awareness of available services and rights may impede service utilization, appropriate referral, and access to support, while misconceptions may reinforce stigma and exclusion, as documented in the literature on intellectual disability [5,7]. Conversely, interpersonal contact with members of a stigmatized group is associated with more favorable attitudes toward that group, a relationship established across contexts by intergroup contact theory [8,9]. Whether disability-related knowledge operates in the same way is less firmly established, and the present study examines awareness and support as distinct dimensions rather than assuming a link between them. Measuring the two dimensions in the same population matters because each implies a different kind of implementation failure, and because the corrective action differs accordingly. Individuals who endorse inclusive policies but are unaware of the support measures enacted under them cannot access or claim those measures, so entitlements remain formally available but practically unused; the constraint here is communication rather than legitimacy. Conversely, individuals who are aware of available services but do not accept the policies underpinning them may not support the allocation of public resources to those services or may not act on that awareness on behalf of others; the constraint is support rather than information. Measuring either dimension alone cannot distinguish these situations, and a policy that is well accepted may still be poorly implemented for want of public knowledge. Examining both within the same population therefore identifies which of the two constraints applies, and in which population groups, which is the level at which communication and education strategies are designed.

Across the Middle East and North Africa, evidence concerning public awareness and attitudes toward disability remains comparatively limited and fragmented. Existing research has frequently focused on specific disability types, parents and caregivers, or healthcare and educational professionals rather than the wider public [10,11]. Studies from the wider Arab region and from international reviews have identified variable levels of disability-related knowledge and persistent misconceptions, particularly regarding developmental conditions such as autism spectrum disorder (ASD) [11,12]; neither of these sources is specific to the Gulf states, which is itself part of the gap this study addresses. Consequently, there remains limited population-level evidence integrating awareness of disability support systems with support for measures intended to promote inclusion.

Saudi Arabia has undergone substantial policy and service development in disability inclusion. National estimates of disability prevalence range from approximately 2.9% to 7.1%, with variation across the country’s thirteen administrative regions [13,14,15]. This range reflects the severity threshold applied rather than disagreement between sources. The 2017 Disability Survey classified functional difficulty according to the Washington Group definition into mild, severe, and extreme degrees, and reported that 7.1% of Saudi citizens experienced at least one functional difficulty of any degree, whereas 2.9% experienced difficulty at the extreme level [14]. An earlier analysis of the 2016 demographic survey, in which disability was recorded as a reported status rather than by graded functional difficulty, estimated prevalence at 3.3% [13]. Saudi Vision 2030 places the empowerment and inclusion of persons with disabilities within the broader national transformation agenda [16]. This commitment has been accompanied by institutional and legislative developments, including the establishment of the Authority for the Care of People with Disabilities and the enactment of the 2023 Law of the Rights of Persons with Disabilities, which strengthened the national framework for protecting rights, facilitating access to services, and promoting participation and empowerment [17]. Specialized services have also expanded, including those for individuals with ASD, although challenges in access and service availability remain [18,19].

Despite these developments, Saudi evidence has largely examined individual disability conditions or specific dimensions of public knowledge and attitudes. Previous studies have reported gaps in public knowledge and familiarity regarding ASD [20,21] and documented stigma experienced by families of children with ASD [22]. More recent work has begun to address the period following these reforms: a 2025 survey of 3353 caregivers of persons with disabilities examined awareness, utilization, and perceived barriers to social and financial services [23], and a 2025 cross-sectional survey of 1007 adults across the five regions assessed public awareness and perceptions of congenital disabilities [24], disability awareness among university graduates [25], and general attitudes toward persons with disabilities [26]. This literature nonetheless remains either condition-specific or focused on caregivers rather than the general public, and it does not examine awareness of support services alongside support for the measures that establish them. Importantly, evidence simultaneously examining public awareness of disability support services and support for disability-inclusive measures remains limited. Understanding both dimensions is particularly relevant as Saudi Arabia moves from establishing disability rights and services toward strengthening their implementation, accessibility, and societal uptake.

Therefore, this study aimed to assess public awareness of disability support services and support for disability-inclusive measures among adults in Saudi Arabia. Specifically, it sought to (1) assess awareness of disability support services, including services for autism spectrum disorder as a condition-specific example; (2) evaluate support for disability-inclusive measures; and (3) identify demographic and disability-exposure factors associated with awareness and support.

## 2. Materials and Methods

### 2.1. Study Design

A cross-sectional online survey was conducted among adults residing across the five geographical regions of Saudi Arabia. The study was designed and reported in accordance with the Strengthening the Reporting of Observational Studies in Epidemiology (STROBE) guidelines for cross-sectional studies [27].

### 2.2. Study Setting and Participants

The study targeted adults residing across the five main geographical regions of Saudi Arabia: Central, Western, Eastern, Southern, and Northern. Participants reported their location by selecting one of these five macro-regions directly. Data were collected between May and July 2026 using a self-administered online questionnaire disseminated through commonly used social media platforms and online community networks, namely WhatsApp, Telegram, X (formerly Twitter), and Facebook. To pursue representation from all five macro-regions rather than relying on a single dissemination channel, data collectors based in each targeted region additionally circulated the questionnaire within their own regional networks.

Individuals were eligible to participate if they were aged ≥ 18 years, currently residing in Saudi Arabia, and able to complete the questionnaire in Arabic. A convenience sampling approach supplemented by snowball dissemination was used, whereby the survey link was circulated through social media networks, and participants were encouraged to share it with eligible individuals within their networks. The questionnaire was administered through Google Forms. All items were set as mandatory, so submitted questionnaires contained no missing data, and the platform was configured to accept a single response per respondent, which limited duplicate submissions. Only responses meeting the eligibility criteria were retained, and a total of 948 responses were included in the final analysis. The number of individuals who opened or began the questionnaire without submitting it was not captured by the platform and therefore cannot be reported, so a response rate cannot be calculated for this survey.

### 2.3. Survey Instrument

A structured questionnaire was developed by the research team. Item content drew on the domains identified in a systematic review of public awareness, attitudes and beliefs regarding intellectual disability [5] and on a Saudi study of public knowledge of ASD [20]; these two sources informed the framing of items rather than providing a validated item pool, and no instrument covering awareness of disability support services alongside support for disability-inclusive measures was available to adapt for the contemporary landscape of disability support services and policies in Saudi Arabia [17]. The questionnaire was originally developed in English and subsequently translated into Arabic by an expert fluent in both languages; the translated version was reviewed for linguistic clarity and consistency, with a large language model (ChatGPT, OpenAI, GPT-6) used as a proofreading aid during refinement, and the final wording was reviewed and approved by the research team. The Arabic version was administered to participants to ensure linguistic accessibility for the target population. Both the English and Arabic versions are provided as Supplementary Material.

For the present study, the questionnaire comprised four analytical components. The first collected demographic and disability-related characteristics, including gender, age group, region of residence, educational level, previous close personal contact with a person with a disability, and the disability type with which participants were most familiar. The questionnaire offered “Yes, friend” as a separate response option for previous close personal contact; for analysis, these responses were combined with “Yes, colleague/classmate” as non-family close contact, and Table 1 reports the combined category. The second comprised six statements addressing awareness and perceptions of disability support services and related public-education needs; following exploratory and confirmatory factor analysis, two of these were retained to compute the composite awareness score, as described in the Statistical Analysis section, while three of the four non-retained items addressed perceptions of public awareness, education needs, and digital support rather than respondents’ own awareness. The fourth non-retained awareness item concerned special accommodations or priority services and did not load with the two service-awareness items. One of the six items referred specifically to services for autism spectrum disorder. This condition was included as a concrete example of condition-specific service awareness because specialized services for autism have expanded visibly in Saudi Arabia and because autism has been the focus of most existing Saudi research on public knowledge of disability [20,21,22]; no other individual condition was assessed separately. The third comprised five candidate items assessing support for disability-inclusive measures. Following factor analysis, four items were retained for the support score; the item stating that disability-related accommodations are fair and necessary was not retained because it did not align clearly with the support factor. Items in the second and third components were rated on a five-point Likert scale ranging from strongly disagree (1) to strongly agree (5). The final component assessed perceived national priorities for disability empowerment using one single-response item and one multiple-response item.

Content validity was assessed by three academic experts with doctoral-level expertise in pharmacy and pharmacoepidemiology, who reviewed the questionnaire for relevance, clarity, and appropriateness. The expert panel did not include specialists in disability studies, rehabilitation, or the social sciences, which is acknowledged as a limitation. The questionnaire was subsequently pilot-tested among 20 adults to assess clarity, comprehensibility, and technical functionality. Following their feedback, minor wording modifications were made to improve clarity and comprehensibility, and technical issues relating to questionnaire administration were identified and corrected; no items were removed. The pilot was intended to evaluate comprehension and functionality rather than to provide formal psychometric validation, and test–retest reliability was not assessed. The 20 pilot participants were not included in the final study sample. Participants involved in the pilot testing were excluded from the final study sample.

Following data collection, the internal structure of the candidate awareness items was examined before calculation of the composite score. The derivation and internal consistency of the final awareness and support scores are described in the Statistical Analysis and Section 3.

### 2.4. Sample Size Calculation

The minimum sample size was initially estimated a priori for multivariable regression analysis using G*Power version 3.1 [28], which required 230 participants (assuming an effect size of f^2^ = 0.15, α = 0.05, power = 95%, and 22 predictors). For the structural equation modeling (SEM) subsequently employed, methodological guidelines generally recommend a minimum sample size of 200 cases or at least 10 observations per estimated parameter. The final sample of 948 participants substantially exceeded both the initial a priori calculation and the recommendations for SEM, ensuring adequate statistical power for the measurement and structural models.

### 2.5. Statistical Analysis

All statistical analyses were performed using R version 4.6.0 with RStudio version 2026.05.1+225 (Posit, Boston, MA, USA) [29]. Statistical significance was defined as a two-sided *p* < 0.05.

Categorical variables were summarized using frequencies and percentages. Composite domain scores were summarized using medians and interquartile ranges (IQRs) because their distributions deviated from normality, as assessed using the Shapiro–Wilk test. Internal consistency was evaluated using Cronbach’s alpha [30]. Item–total correlations were examined when evaluating the internal structure of the awareness and support items.

The internal structure underlying the awareness and support items was examined using exploratory factor analysis (EFA) with Kaiser–Meyer–Olkin (KMO) measures of sampling adequacy and Bartlett’s test of sphericity. Confirmatory factor analysis (CFA) was subsequently conducted to validate the two-factor structure identified by the EFA, using weighted least squares mean and variance adjusted (WLSMV) estimation with theta parameterization to account for the ordinal nature of item responses. Model fit was evaluated using the chi-square test, comparative fit index (CFI), Tucker–Lewis index (TLI), root mean square error of approximation (RMSEA), and standardized root mean square residual (SRMR). Reliability of each latent factor was assessed using Cronbach’s alpha, McDonald’s omega, and average variance extracted (AVE).

The awareness score was derived from two conceptually aligned items assessing self-reported awareness of disability-related support services and autism-related support services. Responses were scored from 1 (strongly disagree) to 5 (strongly agree) and summed up to produce a total score ranging from 2 to 10, with higher scores indicating greater self-reported awareness. The support score comprised of four items assessing support for disability-inclusive measures and services, producing a total score ranging from 4 to 20, with higher scores indicating greater support.

To account for the association between awareness and support for disability-inclusive measures, a structural equation model (SEM) was fitted in which both constructs were specified as latent variables, retaining their CFA-derived measurement structure, and simultaneously regressed on gender, age group, region of residence, educational level, previous personal contact with a person with a disability, and disability type familiarity, with support additionally regressed on awareness. This approach was adopted in place of separate multivariable linear regression models for each domain score, as including each domain score as a predictor of the other in two independent regressions would not account for their reciprocal association and could introduce endogeneity. The SEM was estimated using WLSMV estimation with theta parameterization. Standardized structural coefficients are reported with 95% confidence intervals (CIs) and *p*-values.

## 3. Results

### 3.1. Participant Characteristics

A total of 948 participants were included in the analysis. Of these, 586 (61.8%) were female and 603 (63.6%) were aged 18–29 years. Participants represented all five geographical regions, with the largest proportion residing in the Western region (*n* = 376, 39.7%). Approximately half held a bachelor’s degree (*n* = 490, 51.7%), and 524 (55.3%) reported no close personal contact with a person with a disability.

More than half of participants (*n* = 492, 51.9%) reported no particular familiarity with any of the disability types listed. Among those reporting familiarity, physical disability (*n* = 145, 15.3%) and autism spectrum disorder (ASD; *n* = 143, 15.1%) were the most frequently reported (Table 1).

### 3.2. Awareness of Disability Support Services and Views on Public Information

This section reports two groups of items that should not be read as a single construct: the first items concern respondents’ own self-reported awareness of services and contributed to the composite awareness score, while the remainder record opinions about public awareness in general, the need for education, and digital systems, and did not enter that score. Self-reported awareness varied across the individual items. Overall, 607 participants (64.0%) agreed or strongly agreed that they were aware of disability-related support services available in Saudi Arabia, while 503 (53.1%) reported awareness of autism-related support services. A substantially larger proportion (*n* = 859, 90.6%) recognized that people with disabilities may receive special support or priority services.

**Figure 1:**
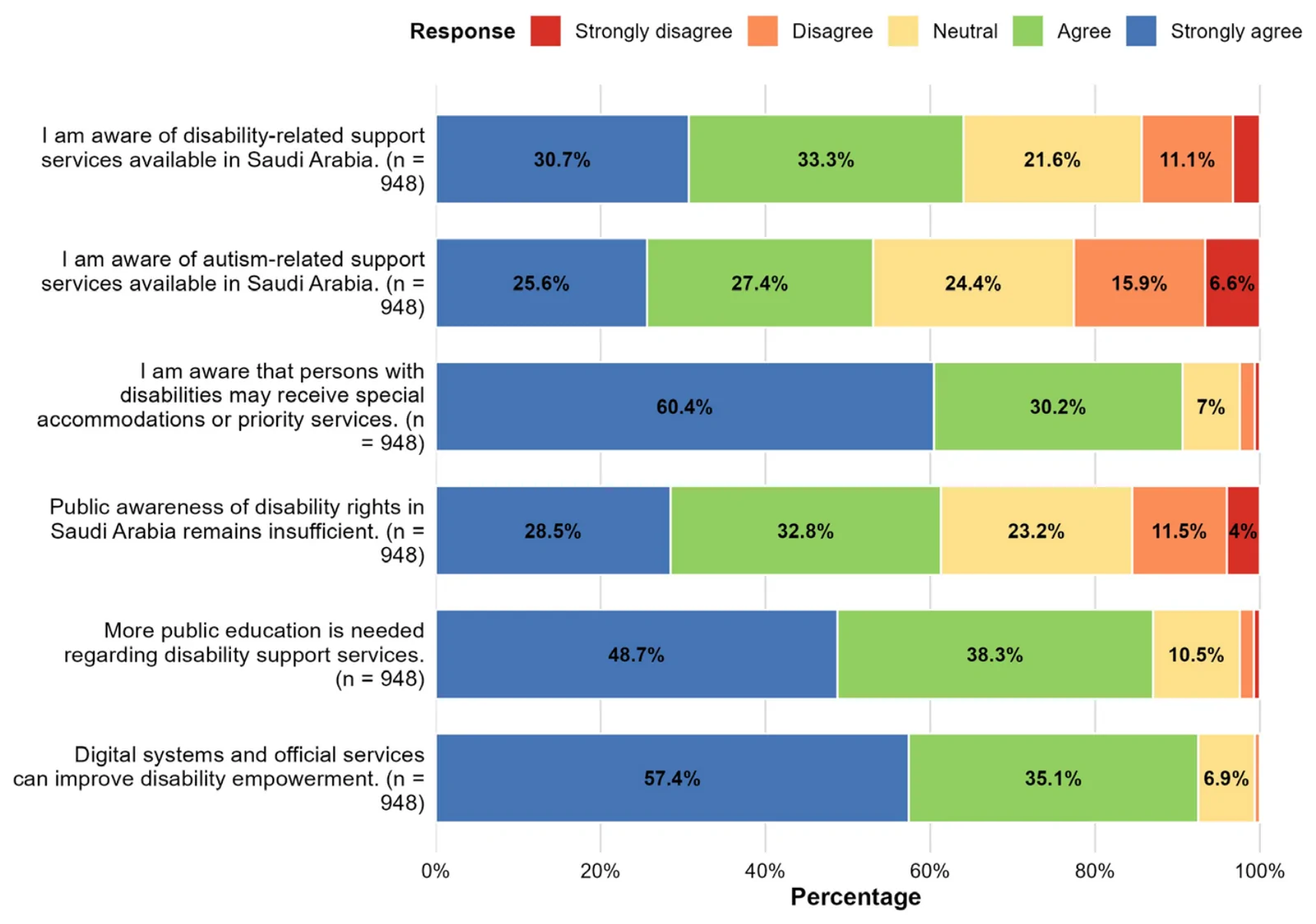
Participants’ responses to items measuring their awareness of disability support services.

Regarding broader perceptions, 581 participants (61.3%) agreed or strongly agreed that public awareness of disability rights in Saudi Arabia remained insufficient, 825 (87.0%) indicated that greater public education regarding disability support services was needed, and 877 (92.5%) agreed that digital systems and official services could improve disability empowerment (Figure 1).

### 3.3. Support for Disability-Inclusive Measures

**Figure 2:**
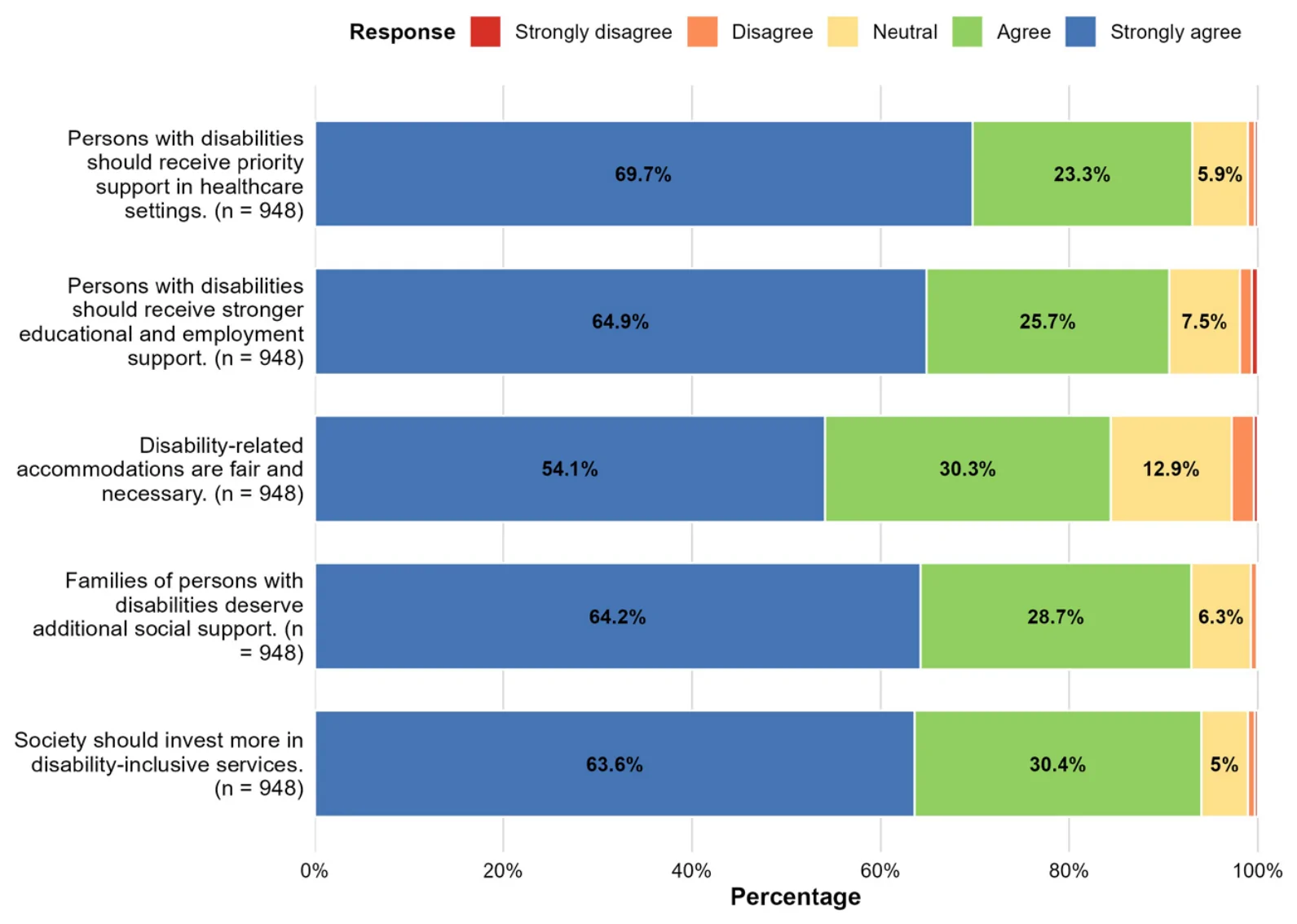
Participants’ responses to items measuring their support for disability-inclusive measures.

Support for disability-inclusive measures was consistently high. A total of 882 participants (93.0%) agreed or strongly agreed that persons with disabilities should receive priority support in healthcare settings, while 859 (90.6%) supported stronger educational and employment support. Furthermore, 800 (84.4%) considered disability-related support measures to be fair and necessary, 881 (92.9%) supported additional social support for families of persons with disabilities, and 891 (94.0%) agreed that society should invest more in disability-inclusive services (Figure 2).

### 3.4. Exploratory Factor Analysis of Awareness and Support Items

Exploratory factor analysis of the eleven candidate items yielded two factors. The overall Kaiser–Meyer–Olkin measure of sampling adequacy was 0.81, and Bartlett’s test of sphericity was significant (χ^2^ = 3841.75, df = 55, *p* < 0.001), indicating the data were suitable for factor analysis. The first factor (MR1) was defined by four items with loadings ranging from 0.73 to 0.74, whereas the second factor (MR2) was defined by two items, with loadings of 0.88 and 0.81. The remaining five items showed weak or cross-loadings (range −0.34 to 0.46) and did not clearly align with either factor (Table 2). The first domain was labelled “Support for Disability-Inclusive Measures” and the second domain was labelled “Awareness of Disability Support Services”.

### 3.5. Confirmatory Factor Analysis of Awareness and Support Constructs

Confirmatory factor analysis supported the two-factor structure identified in the exploratory analysis. Model fit was excellent (χ^2^(8) = 60.93, *p* < 0.001; CFI = 0.998; TLI = 0.997; RMSEA = 0.054, 90% CI 0.035 to 0.076; SRMR = 0.025). The Support for Disability-Inclusive Measures factor was defined by four items with standardized loadings ranging from 0.839 to 0.863 (all *p* < 0.001), demonstrating good internal consistency (Cronbach’s alpha = 0.845, McDonald’s omega = 0.866) and adequate convergent validity (AVE = 0.735). The Awareness of Disability Support Services factor was defined by two items with standardized loadings of 0.995 and 0.807 (*p* < 0.001), also demonstrating good reliability (Cronbach’s alpha = 0.846, McDonald’s omega = 0.860, AVE = 0.820; Table 3).

### 3.6. Item Reliability Analysis of Awareness and Support Scales

Item–total correlations for the Awareness of Disability Support Services scale were high, ranging from 0.924 to 0.939, with strong inter-item correlation (r = 0.736) and a Spearman–Brown coefficient of 0.848, indicating reliable measurement with the two-item scale. For the Support for Disability-Inclusive Measures scale, item–total correlations ranged from 0.811 to 0.849, and reliability coefficients with each item deleted ranged from 0.798 to 0.812, indicating consistent contribution of each item to the overall scale (Table 4).

The Awareness of Disability Support Services score (2 items) had a median of 8.0 (Q1–Q3: 6.0–9.0), ranging from 2.0 to 10.0, and the Support for Disability-Inclusive Measures score (4 items) had a median of 19.0 (Q1–Q3: 17.0–20.0), ranging from 7.0 to 20.0; both scores deviated significantly from normality (Shapiro–Wilk *p* < 0.001 for both).

### 3.7. Structural Equation Modeling of Awareness and Support

The structural model demonstrated excellent fit to the data: scaled χ^2^(92) = 139.18, *p* = 0.001; CFI = 0.993; TLI = 0.999; RMSEA = 0.023 (90% CI [0.015, 0.031]); SRMR = 0.018; and explained 16.6% of the variance in support for disability-inclusive measures and 12.1% in awareness. Awareness was positively associated with support for disability-inclusive measures (β = 0.299, 95% CI 0.225 to 0.372, *p* < 0.001). Support was also higher among participants residing in the Western region (β = 0.131, 95% CI 0.029 to 0.232, *p* = 0.011), those with secondary-school education (β = 0.183, 95% CI 0.024 to 0.343, *p* = 0.024), and those with a bachelor’s degree (β = 0.244, 95% CI 0.072 to 0.416, *p* = 0.005), compared with primary-school education. Support was also higher among those with a diploma (β = 0.119, 95% CI 0.002 to 0.235, *p* = 0.046), compared with primary-school education. Support was lower among participants reporting no prior personal contact with a person with a disability (β = −0.184, 95% CI −0.313 to −0.055, *p* = 0.005) and among those unsure of such contact (β = −0.117, 95% CI −0.193 to −0.041, *p* = 0.003), compared with those reporting contact with a family member, and among those most familiar with hearing impairment (β = −0.086, 95% CI −0.161 to −0.011, *p* = 0.024), compared with those familiar with no disability type.

For Awareness, scores were lower among participants residing in the Eastern region (β = −0.129, 95% CI −0.203 to −0.055, *p* < 0.001) and Northern region (β = −0.146, 95% CI −0.217 to −0.075, *p* < 0.001), compared with the Central region, and scores were higher among those residing in the Southern region (β = 0.112, 95% CI 0.017 to 0.206, *p* = 0.020), compared with the Central region. Awareness was lower among participants with a bachelor’s degree (β = −0.312, 95% CI −0.506 to −0.117, *p* = 0.002), postgraduate degree (β = −0.181, 95% CI −0.297 to −0.065, *p* = 0.002), and secondary-school education (β = −0.221, 95% CI −0.400 to −0.042, *p* = 0.015), compared with primary-school education. Awareness was higher among those aged 30–39 years (β = 0.089, 95% CI 0.017 to 0.161, *p* = 0.015), compared with those aged 18–29 years (Table 5).

### 3.8. Public Priorities for Disability Empowerment

When asked to identify the single highest national priority for improving disability inclusion, 496 participants (52.3%) considered all listed areas equally important. Among participants selecting a specific priority, public awareness and education was the most frequently selected (*n* = 204, 21.5%), followed by healthcare accessibility (*n* = 105, 11.1%).

When multiple approaches could be selected, public awareness campaigns were most frequently endorsed (*n* = 715, 75.4%), followed by school-based education (*n* = 626, 66.0%), family and community programmes (*n* = 619, 65.3%), and healthcare professional training (*n* = 508, 53.6%) (Table 6).

## 4. Discussion

This cross-sectional online survey examined public awareness of disability support services and support for disability-inclusive measures among 948 adults across all five geographical regions of Saudi Arabia. The findings reveal a notable contrast: self-reported awareness of available support services was reported by roughly two-thirds of participants for general disability services and by around half for autism-specific services, whereas support for disability-inclusive measures was endorsed by more than 84% of participants across every item. Awareness varied significantly by region, age, and education: compared with the Central region, awareness was lower in the Eastern and Northern regions and higher in the Southern region; it was higher among participants aged 30–39 years than among those aged 18–29 years and lower among those with secondary-school, bachelor’s, or postgraduate education than among those with primary-school education. Support for disability-inclusive measures was higher among participants in the Western region and among those with secondary-school, diploma, or bachelor’s education, and lower among those reporting no or uncertain previous personal contact with a person with a disability and among those most familiar with hearing impairment; the female–male difference was not statistically significant. Importantly, awareness and support for disability-inclusive measures were positively associated, while public awareness campaigns emerged as the most frequently endorsed strategy for improving disability inclusion. Collectively, these findings suggest broad societal support for disability inclusion alongside uneven public awareness of the services through which such policies are implemented.

### 4.1. Awareness of Disability Support Services

Approximately two-thirds of participants reported awareness of general disability-related support services, while just over half reported awareness of autism-related support services. These findings suggest that information about available services has not reached all segments of the population. This pattern is broadly consistent with previous Saudi studies reporting limited objectively assessed knowledge of ASD among the general population [20] and insufficient public familiarity with autism [21]. Importantly, however, the present study assessed self-reported awareness rather than objectively tested knowledge. Self-assessment and objectively measured knowledge represent distinct constructs and may not necessarily correspond [31]. The comparatively lower awareness of autism-specific services than of general disability services may also reflect differences in the public visibility and availability of specialized services, particularly given previous evidence of geographical variation in ASD service provision [18].

Significant regional differences were observed after adjustment for other measured characteristics. Compared with participants in the Central region, awareness scores were lower among those in the Eastern and Northern regions. Awareness was also higher among participants in the Southern region than among those in the Central region. Previous national evidence has demonstrated geographical variation in disability prevalence and service distribution [13,14], while specialized disability and ASD services may also vary geographically [18]. However, the observed pattern does not support a simple relationship between presumed service availability and public awareness. Local communication channels, community engagement, and exposure to disability organizations are plausible contributors, but none of these were measured here, and the present data cannot adjudicate between them; they are stated as hypotheses for future work rather than as explanations. Two further cautions apply to the regional findings themselves. The sample was recruited by convenience and snowball dissemination and was unevenly distributed across regions, so regional groups differ in size and in how they were reached. Region of residence was self-reported and not independently verified. Regional comparisons should therefore be read as descriptive of this sample rather than as estimates of regional differences in the population.

An unexpected finding was the inverse association between educational attainment and self-reported awareness. Compared with participants whose highest attainment was primary school, awareness was lower among those with secondary-school, bachelor’s, and postgraduate education, while the diploma estimate did not reach statistical significance. The pattern was therefore not monotonic across the full educational range. Awareness was also higher among participants aged 30–39 years than among those aged 18–29 years; other age-group comparisons were not statistically significant. Two qualifications apply. First, the reference category comprised only 37 participants (3.9% of the sample), which makes these estimates sensitive to the mean of that small group and correspondingly imprecise. Second, the pattern for support ran in the opposite direction, with secondary-school, diploma, and bachelor’s-degree participants reporting greater support than the reference group, so educational attainment is not associated uniformly with both outcomes. This contrasts with the conventional expectation that greater educational attainment accompanies better access to health information; this expectation rests on work concerning health literacy and socioeconomic status [32] rather than on evidence about awareness of disability support services specifically. Several explanations are possible, although they remain speculative. More highly educated respondents may apply stricter criteria when evaluating their own awareness, resulting in more conservative self-assessments; discrepancies between perceived and objectively measured competence have been documented in other contexts [31]. Social and structural factors may also contribute. Proximity to a service system shapes awareness of it as much as general education does: participants with lower educational attainment may on average have more direct contact with publicly funded disability support, whether through eligibility for assistance or greater reliance on public provision, whereas those with higher attainment may obtain comparable support privately or have less occasion to seek it. The information channels through which each group encounters service-related material may differ in the same way. These interpretations were not tested in the present data and are offered as hypotheses for future work. Although the analysis adjusted for age, region, personal disability contact, and other measured characteristics, residual confounding remains possible. This finding should therefore be interpreted cautiously and replicated using objective measures of service knowledge before informing targeted interventions.

### 4.2. Support for Disability-Inclusive Measures

Public support for disability-inclusive measures was consistently high, with agreement or strong agreement ranging from 84.4% to 94.0% across individual items. Particularly strong support was observed for investment in disability-inclusive services, priority healthcare support, and additional social support for families of people with disabilities. Agreement at this level produces a pronounced ceiling effect: because all items were positively worded and few respondents disagreed with any of them, the scale distinguishes poorly between degrees of support, and the associations reported below are estimated over a narrow range of variation. Items of this kind are also among the most exposed to social desirability, since disagreement would require opposing provisions for a group widely regarded as deserving of them. These findings are broadly consistent with the increasing prominence of disability rights, empowerment, and social participation within Saudi Arabia’s national reform agenda [16,17]. What these items establish is that respondents endorsed the principle of these provisions when asked directly. They do not establish support for any particular policy instrument, nor willingness to accept its costs or trade-offs, and high stated support should not necessarily be interpreted as equivalent to behavioral support or effective implementation.

Support scores were slightly higher among female than male participants, but this difference did not reach statistical significance in the structural model. Gender differences in attitudes toward people with disabilities have been reported across settings [6,33]; those studies measured attitudes rather than support for specific measures, and the present data neither confirm nor exclude a difference in the latter. Support was higher among participants in the Western region than among those in the Central region; this regional association should be interpreted cautiously for the same sampling reasons noted above. Participants with secondary-school education, a diploma, or a bachelor’s degree demonstrated greater support than those with primary-school education, whereas postgraduate education was not independently associated with support. This distinction is important because the findings do not establish a general dose–response relationship between education and support.

Participants who reported no previous close personal contact with a person with a disability, as well as those who were unsure whether such contact had occurred, reported lower support than those reporting contact with a family member. No statistically significant adjusted difference was observed for participants reporting contact with a colleague or classmate. Support was also lower among participants most familiar with hearing impairment than among those reporting familiarity with no disability type. Because the hearing-impairment subgroup was small (*n* = 38), this association should be interpreted cautiously and warrants replication. Although meaningful interpersonal contact has been associated with more favorable attitudes toward stigmatized groups and is consistent with intergroup contact theory [8,9], the present findings provide only limited support for such an interpretation. In particular, the relatively small “unsure” subgroup and the observational nature of the contact measure warrant cautious interpretation.

### 4.3. Relationship Between Awareness and Support

One of the most notable findings was the positive association between awareness of disability support services and support for disability-inclusive measures. In the structural equation model, awareness was positively associated with support for disability-inclusive measures after adjustment for demographic characteristics and disability exposure (β = 0.299, 95% CI 0.225 to 0.372, *p* < 0.001). The model specified this path in one direction only, so the estimate should not be read as evidence that awareness produces support. Because awareness and support were estimated within the same structural model, this result should be interpreted as a conditional association rather than as evidence of a causal or reciprocal effect.

The cross-sectional design precludes determining the direction of this relationship. Greater awareness may contribute to more favorable attitudes toward disability-inclusive measures; conversely, individuals who already hold favorable attitudes may be more likely to seek, retain, or engage with information about disability services. Both may also be influenced by unmeasured factors such as prior exposure to disability advocacy, media content, or community engagement. The findings are therefore best interpreted as evidence of an association between awareness and support for disability-inclusive measures rather than a causal pathway.

### 4.4. Public Priorities for Disability Empowerment

Participants stated priorities provide additional context for interpreting these findings. More than half (52.3%) considered all listed areas—public awareness and education, healthcare accessibility, educational inclusion, employment opportunities, and family support—to be equally important national priorities. When specific strategies for improving disability inclusion were considered, public awareness campaigns were the most frequently endorsed approach (75.4%), followed by school-based education (66.0%) and family and community programmes (65.3%). These priorities are consistent with the finding that 61.3% of respondents considered current public awareness of disability rights to be insufficient.

Furthermore, 92.5% agreed that digital systems and official services could improve disability empowerment. This figure records what participants considered promising, not whether digital channels improve awareness in practice, which this study did not test. Read that way, it indicates public receptiveness to digital dissemination rather than evidence of its effectiveness. However, the present study assessed public perceptions of the potential value of digital systems rather than their actual use or effectiveness. Future intervention studies should therefore evaluate whether digital awareness strategies improve objective knowledge, service navigation, and uptake.

Taken together, the findings identify where self-reported awareness was lowest and record which strategies participants endorsed. They do not establish that any of those strategies would raise awareness, since none was tested here. Regionally responsive communication, school- and community-based education, and accessible digital information are therefore offered as candidates for evaluation alongside ongoing disability policy development under Saudi Vision 2030 [16], not as measures shown to be effective.

### 4.5. Strengths and Limitations

This study has several strengths. It included a relatively large sample of 948 adults with participation from all five geographical regions of Saudi Arabia and simultaneously examined two complementary dimensions of disability inclusion: awareness of available support services and support for disability-inclusive measures. The analysis also moved beyond descriptive reporting by examining the directional association from awareness to support for disability-inclusive measures. Using a structural equation model (SEM) with weighted least squares mean and variance adjusted (WLSMV) estimation.

Several limitations should nevertheless be considered. First, the two central constructs were defined operationally rather than drawn from an established theoretical framework, and the items measure self-reported awareness and endorsement of support measures rather than tested knowledge or acceptance of named policies; readers should hold this in mind when comparing these findings with studies using different definitions. The content-validity process was correspondingly limited: three experts reviewed the instrument, none of whom worked in disability studies, rehabilitation, disability policy or psychometrics, and no existing validated instrument was adapted. Second, the cross-sectional design precludes causal inference, particularly regarding the relationship between awareness and support for disability-inclusive measures. Second, online convenience sampling with snowball dissemination may have introduced selection bias [34]. The survey platform did not capture the number of individuals who opened or began the questionnaire, so a response rate cannot be calculated, and the extent of non-response bias cannot be quantified. Younger adults, females, participants from the Western region, and those with a bachelor’s degree constituted substantial proportions of the sample; consequently, the findings should not be interpreted as nationally representative despite participation from all five regions.

Third, all measures were self-reported and may therefore be susceptible to social desirability and response biases [35]. The high endorsement of disability-inclusive measures may partly reflect socially desirable responding, and no dedicated social-desirability measure was included. The concentration of responses toward the upper end of the support scale may also have reduced variability and limited the ability to identify additional correlates.

Fourth, awareness was assessed through respondents’ perceptions of their own awareness rather than objective testing of their knowledge of specific services, eligibility requirements, or access pathways. The findings should therefore be interpreted as self-reported awareness, not demonstrated service knowledge.

Fifth, the questionnaire was developed for this study rather than adapted from a previously validated instrument. Its structure was examined through exploratory and confirmatory factor analysis, which supported a two-factor solution with good fit and acceptable reliability for both constructs, and the composite awareness score retains the two items that loaded clearly on the awareness factor. This establishes structural validity within the present sample but does not constitute full psychometric validation: the instrument has not been evaluated in an independent sample, test–retest reliability was not assessed, and no external criterion was available against which to examine convergent validity. The two-item awareness score in particular rests on a small number of indicators, and independent evaluation is warranted before the instrument is used elsewhere. Finally, the structural model explained a modest proportion of outcome variance (12.1% for awareness and 16.6% for support for disability-inclusive measures), suggesting that potentially important determinants—including media exposure, socioeconomic status, occupational experience, health literacy, and engagement with disability services—were not captured.

### 4.6. Future Directions

This study joins a small but growing Saudi evidence base that includes work on disability awareness among university graduates [25] and on attitudes toward and communication with persons with disabilities [26]; what remains largely unexamined is how awareness of services and support for the measures that fund them relate to one another in the general adult population, which is the question taken up here. Future studies should validate these findings using probability-based or otherwise more representative sampling approaches and incorporate objective measures of knowledge alongside self-reported awareness. Longitudinal and quasi-experimental studies could evaluate whether targeted awareness interventions produce measurable improvements in knowledge, attitudes, service navigation, and service utilization. The regional differences identified here also warrant further investigation to determine whether they reflect variation in service availability, information dissemination, community engagement, or other contextual factors. Importantly, future research should incorporate the perspectives of persons with disabilities and their families to complement the general-public perspective and ensure that proposed inclusion strategies reflect lived experience and actual service needs.

## 5. Conclusions

Adults participating in this cross-sectional online survey endorsed disability-inclusive support measures at a high rate, while self-reported awareness of disability and autism-related support services was more variable across population groups. What was measured was agreement with the principle of such measures rather than acceptance of any specific policy. Awareness and support for disability-inclusive measures were positively associated, and participants identified public awareness campaigns, school-based education, and community-based initiatives as important strategies for strengthening disability inclusion. These findings suggest that continued expansion of disability-support systems should be accompanied by accessible and regionally responsive communication strategies that improve public understanding of available services. Strengthening the connection between established disability policies and public knowledge may support broader implementation of disability inclusion and empowerment objectives under Saudi Vision 2030.

## Figures and Tables

**Table 1:** Demographic characteristics and familiarity with disability.

Characteristic	Description
Gender	
Male	362 (38.2%)
Female	586 (61.8%)
Age	
18–29 years	603 (63.6%)
30–39 years	113 (11.9%)
40–49 years	136 (14.3%)
50–59 years	69 (7.3%)
60 years or older	27 (2.8%)
Region of Residence	
Central	212 (22.4%)
Western	376 (39.7%)
Eastern	109 (11.5%)
Southern	165 (17.4%)
Northern	86 (9.1%)
Highest Educational Level	
Primary school	37 (3.9%)
Secondary school	278 (29.3%)
Diploma	82 (8.6%)
Bachelor’s degree	490 (51.7%)
Postgraduate degree (Master’s or Doctorate level)	61 (6.4%)
Ever had close personal contact with a person with a disability	
Yes, family member	255 (26.9%)
Yes, friend/colleague/classmate	98 (10.3%)
No	524 (55.3%)
Unsure	71 (7.5%)
Disability type which you are most familiar with	
None	492 (51.9%)
Autism Spectrum Disorder (ASD)	143 (15.1%)
Intellectual disability	89 (9.4%)
Visual impairment	41 (4.3%)
Hearing impairment	38 (4.0%)
Physical disability	145 (15.3%)
*n* (%)	

**Table 2:** Exploratory factor analysis: individual Kaiser–Meyer–Olkin (KMO) measures of sampling adequacy and rotated factor loadings for each item.

MR2	MR1	Individual KMO	Variable
	0.74	0.87	Society should invest more in disability-inclusive services.
	0.74	0.86	Families of persons with disabilities deserve additional social support.
	0.73	0.86	Persons with disabilities should receive priority support in healthcare settings.
	0.73	0.86	Persons with disabilities should receive stronger educational and employment support.
	0.31	0.86	Digital systems and official services can improve disability empowerment.
	−0.23	0.75	More public education is needed regarding disability support services.
	0.46	0.93	Disability-related accommodations are fair and necessary.
	0.39	0.86	I am aware that persons with disabilities may receive special accommodations or priority services.
	−0.34	0.67	Public awareness of disability rights in Saudi Arabia remains insufficient.
0.88		0.61	I am aware of autism-related support services available in Saudi Arabia.
0.81		0.64	I am aware of disability-related support services available in Saudi Arabia.

Overall KMO MSA = 0.81|Bartlett’s Test of Sphericity: Chi-Square = 3841.75, df = 55, p-value ≤ 0.001.

**Table 3:** Confirmatory factor analysis: standardized factor loadings and reliability estimates (Cronbach’s alpha, McDonald’s omega, and average variance extracted) for each latent factor.

	Reliability		Factor Loadings		Variable Label	Domain
AVE	McDonald Omega	Cronbach’s Alpha	*p* Value	Standardized Values		
0.735	0.866	0.845		0.839	Persons with disabilities should receive priority support in healthcare settings.	Support for Disability-Inclusive Measures
			<0.001	0.863	Persons with disabilities should receive stronger educational and employment support.	
			<0.001	0.861	Families of persons with disabilities deserve additional social support.	
			<0.001	0.861	Society should invest more in disability-inclusive services.	
0.82	0.86	0.846		0.995	I am aware of disability-related support services available in Saudi Arabia.	Awareness of Disability Support Services
			<0.001	0.807	I am aware of autism-related support services available in Saudi Arabia.	

CFA is based on a total of 948 observations. Fit indicators: χ^2^(8) = 60.93, *p* < 0.001, CFI = 0.998, TLI = 0.997, RMSEA = 0.054 (90% CI, 0.035 to 0.076), and SRMR = 0.025.

**Table 4:** Item–total correlations and reliability coefficients with each item deleted for the Awareness and Support scales.

Reliability if an Item Is Dropped	Item–Total Correlation	Labels	Variable
0.665	0.924	I am aware of disability-related support services available in Saudi Arabia.	Awareness of Disability Support Services
0.815	0.939	I am aware of autism-related support services available in Saudi Arabia.	
0.812	0.811	Persons with disabilities should receive priority support in healthcare settings.	Support for Disability-Inclusive Measures
0.798	0.849	Persons with disabilities should receive stronger educational and employment support.	
0.801	0.826	Families of persons with disabilities deserve additional social support.	
0.804	0.822	Society should invest more in disability-inclusive services.	

**Table 5:** Structural equation model: factors associated with Awareness and Support, examining awareness as a predictor of support.

Outcome	Predictor	β (Std.)	95% CI	*p*-Value
Support	Awareness (latent)	0.299	0.225, 0.372	<0.001
	Gender: Female	0.066	−0.014, 0.146	0.106
	Age: 30 39 years	−0.016	−0.093, 0.060	0.678
	Age: 40 49 years	0.050	−0.039, 0.140	0.270
	Age: 50 59 years	0.072	−0.010, 0.154	0.085
	Age: 60 years or older	0.024	−0.061, 0.108	0.586
	Region: Western	0.131	0.029, 0.232	0.011
	Region: Eastern	0.018	−0.067, 0.103	0.678
	Region: Southern	0.049	−0.048, 0.147	0.322
	Region: Northern	0.021	−0.060, 0.102	0.610
	Education: secondary school	0.183	0.024, 0.343	0.024
	Education: diploma	0.119	0.002, 0.235	0.046
	Education: Bachelor s degree	0.244	0.072, 0.416	0.005
	Education: postgraduate degree master s or doctorate level	0.046	−0.070, 0.163	0.435
	Contact: yes colleague classmate	−0.047	−0.126, 0.033	0.252
	Contact: no	−0.184	−0.313, −0.055	0.005
	Contact: unsure	−0.117	−0.193, −0.041	0.003
	Disability type: autism spectrum disorder	−0.009	−0.114, 0.095	0.858
	Disability type: intellectual disability	−0.085	−0.176, 0.007	0.071
	Disability type: visual impairment	0.023	−0.064, 0.110	0.606
	Disability type: hearing impairment	−0.086	−0.161, −0.011	0.024
	Disability type: physical disability	−0.010	−0.112, 0.092	0.845
Awareness	Gender: Female	0.037	−0.037, 0.111	0.324
	Age: 30 39 years	0.089	0.017, 0.161	0.015
	Age: 40 49 years	0.003	−0.071, 0.078	0.928
	Age: 50 59 years	0.067	−0.006, 0.140	0.071
	Age: 60 years or older	−0.038	−0.130, 0.054	0.417
	Region: Western	−0.015	−0.110, 0.080	0.761
	Region: Eastern	−0.129	−0.203, −0.055	<0.001
	Region: Southern	0.112	0.017, 0.206	0.020
	Region: Northern	−0.146	−0.217, −0.075	<0.001
	Education: secondary school	−0.221	−0.400, −0.042	0.015
	Education: diploma	−0.120	−0.247, 0.007	0.065
	Education: Bachelor s degree	−0.312	−0.506, −0.117	0.002
	Education: postgraduate degree master s or doctorate level	−0.181	−0.297, −0.065	0.002
	Contact: yes colleague classmate	0.038	−0.040, 0.116	0.344
	Contact: no	−0.082	−0.192, 0.028	0.146
	Contact: unsure	−0.024	−0.103, 0.055	0.558
	Disability type: autism spectrum disorder	0.067	−0.018, 0.151	0.121
	Disability type: intellectual disability	0.028	−0.058, 0.115	0.519
	Disability type: visual impairment	−0.047	−0.119, 0.024	0.196
	Disability type: hearing impairment	−0.038	−0.115, 0.038	0.329
	Disability type: physical disability	−0.070	−0.163, 0.023	0.139

Reference categories: male; 18–29 years; Central region; primary school; family member contact; none. The structural equation model demonstrated excellent fit to the data: scaled χ^2^(92) = 139.18, *p* = 0.001; scaled CFI = 0.993; scaled TLI = 0.999; scaled RMSEA = 0.023 (90% CI [0.015, 0.031]); and SRMR = 0.018.

**Table 6:** Participants’ responses regarding their perceptions about public priorities for disability empowerment.

Characteristic	Description
Area which should be the highest national priority for improving disability inclusion	*n* (%)
Public awareness and education	204 (21.5%)
Healthcare accessibility	105 (11.1%)
Educational inclusion	45 (4.7%)
Employment opportunities	62 (6.5%)
Family support services	36 (3.8%)
All equally important	496 (52.3%)
Approaches that would most effectively improve disability inclusion *	*n* (%)
Family/community programs	619 (65.3%)
Government policy reforms	303 (32.0%)
Media representation	411 (43.4%)
Healthcare professional training	508 (53.6%)
Public awareness campaigns	715 (75.4%)
School education	626 (66.0%)

* A multiple-response item.

## Data Availability

The dataset used and analyzed during the current study is available from the corresponding author upon reasonable request. The data are not publicly available due to ethical and confidentiality considerations related to the conditions under which participant consent and ethical approval were obtained.

## Supplementary Materials

The following supporting information can be downloaded at: https://www.mdpi.com/article/10.3390/healthcare14213666/s1, Supplementary Material: Public awareness of disability support services and social inclusion attitudes toward autism and other disabilities.

## Abbreviations

The following abbreviations are used in this manuscript:

ASD	autism spectrum disorder
AVE	average variance extracted
CFA	confirmatory factor analysis
CFI	comparative fit index
CI	confidence interval
EFA	exploratory factor analysis
IQR	interquartile range
KMO	Kaiser–Meyer–Olkin
MSA	measure of sampling adequacy
RMSEA	root mean square error of approximation
SEM	structural equation model
SRMR	standardized root mean square residual
STROBE	Strengthening the Reporting of Observational Studies in Epidemiology
TLI	Tucker–Lewis index
WLSMV	weighted least squares mean and variance adjusted
