# Supplementary material for: Public Awareness of Disability Support Services and Support for Disability-Inclusive Measures in Saudi Arabia: A Cross-Sectional Online Survey
Source: Healthcare (Basel). 2026 Oct 28;14(21):3666. doi: 10.3390/healthcare14213666 (PMC13762876; doi:10.3390/healthcare14213666)
Supplement: Supplementary file 1 [file healthcare-14-03666-s001.zip › healthcare-4540110-supplementary.pdf]

<u>Study Questionnaire</u>

Title: Public Awareness of Disability Support Services and Support for Disability-Inclusive Measures in Saudi Arabia: A Cross-Sectional Online Survey

Description:

- The aim of this study is to assess public awareness of disability support services and social inclusion attitudes toward individuals with autism and other disabilities in Saudi Arabia.
- Participation is voluntary, anonymous, and confidential.
- Estimated completion time: 5–6 minutes.

Section 1: Consent

1. Do you agree to participate in this study?

Yes, I agree to participate

No, I do not agree

(If "No" → End survey)

Section 2: Eligibility screening

2. Are you currently living in Saudi Arabia?

Yes

No

(If "No" → End survey)

3. Are you 18 years of age or older?

Yes

No

(If "No" → End survey)

Section 3: Demographics

4. Age:

18–29 years

30–39 years

40–49 years

50–59 years

60 years or older

5. Gender:

Male

Female

6. Region of living:

Central

Western

Eastern

Southern

Northern

7. Highest educational level:

Primary school

Secondary school

Diploma

Bachelor’s degree

Postgraduate degree (Master’s or Doctorate level)

8. Have you ever had close personal contact with a person with a disability?

Yes, family member

Yes, friend

Yes, colleague/classmate

No

Unsure

9. Which disability type are you most familiar with?

Autism Spectrum Disorder (ASD)

Intellectual disability

Visual impairment

Hearing impairment

Physical disability

None

Section 4: Awareness of Disability Support Services

Response options:

Response options: Strongly agree | Agree | Neutral | Disagree | Strongly disagree

10. I am aware of disability-related support services available in Saudi Arabia.

11. I am aware of autism-related support services available in Saudi Arabia.

12. I am aware that persons with disabilities may receive special accommodations or priority services.

13. Public awareness of disability rights in Saudi Arabia remains insufficient.

14. More public education is needed regarding disability support services.

15. Digital systems and official services can improve disability empowerment.

Section 5: Acceptance of Disability Support Policies

Response options:

Response options: Strongly agree | Agree | Neutral | Disagree | Strongly disagree

16. Persons with disabilities should receive priority support in healthcare settings.

17. Persons with disabilities should receive stronger educational and employment support.

18. Disability-related accommodations are fair and necessary.

19. Families of persons with disabilities deserve additional social support.

20. Society should invest more in disability-inclusive services.

Section 8: Public Priorities for Disability Empowerment

29. Which area should be the highest national priority for improving disability inclusion?

Public awareness and education

Healthcare accessibility

Educational inclusion

Employment opportunities

Family support services

All equally important

30. Which approaches would most effectively improve disability inclusion? (Select all that apply)

Public awareness campaigns

School education

Healthcare professional training

Government policy reforms

Media representation

Family/community programs

End Of Survey:

Thank you for your participation. Your responses will help strengthen disability awareness, social inclusion, and empowerment strategies for persons with disabilities in Saudi Arabia.

العنوان : وعي المجتمع بخدمات دعم الأشخاص ذوي الإعاقة وتأييد التدابير الداعمة لهم في المملكة العربية السعودية :دراسة مقطعية

وصف الاستبيان:

- تهدف هذه الدراسة إلى تقييم مستوى الوعي المجتمعي بخدمات دعم الأشخاص ذوي الإعاقة، واتجاهات الدمج الاجتماعي تجاه الأشخاص ذوي اضطراب طيف التوحد والإعاقات الأخرى في المملكة العربية السعودية.
- المشاركة في هذه الدراسة طوعية ومجهولة الهوية، مع ضمان سرية جميع الإجابات.
- الوقت المتوقع لإكمال الاستبيان 6–5 :دقائق.

القسم :1الموافقة على المشاركة

.1هل توافق على المشاركة في هذه الدراسة؟

نعم، أوافق على المشاركة

لا، لا أوافق على المشاركة

)إذا كانت الإجابة »لا«، يُنهى الاستبيان(

القسم :2التحقق من الأهلية

.2هل تقيم حاليًا في المملكة العربية السعودية؟

نعم

لا

)إذا كانت الإجابة »لا«، يُنهى الاستبيان(

.3هل عمرك 18سنة أو أكثر؟

نعم

لا

)إذا كانت الإجابة »لا«، يُنهى الاستبيان(

القسم :3المعلومات العامة

.4العمر:

29–18سنة

39–30سنة

40–49سنة

50–59سنة

60سنة أو أكثر

.5الجنس:

ذكر

أنثى

.6منطقة السكن:

المنطقة الوسطى

المنطقة الغربية

المنطقة الشرقية

المنطقة الجنوبية

المنطقة الشمالية

.7أعلى مستوى تعليمي:

المرحلة الابتدائية

المرحلة الثانوية

دبلوم

درجة البكالوريوس

الدراسات العليا)ماجستير أو دكتوراه(

.8هل سبق لك أن كان لديك تواصل شخصي وثيق مع أحد الأشخاص ذوي الإعاقة؟

نعم، أحد أفراد العائلة

نعم، صديق

نعم، زميل في العمل أو الدراسة

لا

غير متأكد

.9ما نوع الإعاقة الذي لديك معرفة أكبر به؟

اضطراب طيف التوحد

الإعاقة الذهنية

الإعاقة البصرية

الإعاقة السمعية

الإعاقة الجسدية

لا يوجد

القسم :4الوعي بخدمات دعم الأشخاص ذوي الإعاقة

خيارات الإجابة:

أوافق بشدة |أوافق |محايد |لا أوافق |لا أوافق بشدة

.10لدي معرفة بخدمات الدعم المتاحة للأشخاص ذوي الإعاقة في المملكة العربية السعودية.

.11لدي معرفة بخدمات الدعم المتاحة للأشخاص ذوي اضطراب طيف التوحد في المملكة العربية السعودية.

.12أعلم أن الأشخاص ذوي الإعاقة قد يحصلون على تسهيلات خاصة أو خدمات ذات أولوية.

.13لا يزال الوعي المجتمعي بحقوق الأشخاص ذوي الإعاقة في المملكة العربية السعودية غير كافٍ.

.14هناك حاجة إلى مزيد من التوعية المجتمعية بشأن خدمات دعم الأشخاص ذوي الإعاقة.

.15يمكن للأنظمة الرقمية والخدمات الرسمية أن تسهم في تمكين الأشخاص ذوي الإعاقة.

القسم :5تقبّل سياسات دعم الأشخاص ذوي الإعاقة

خيارات الإجابة:

أوافق بشدة |أوافق |محايد |لا أوافق |لا أوافق بشدة

.16يجب أن يحصل الأشخاص ذوو الإعاقة على أولوية الدعم في خدمات الرعاية الصحية.

.17يجب أن يحصل الأشخاص ذوو الإعاقة على دعم أكبر في التعليم وفرص العمل.

.18التسهيلات المقدمة للأشخاص ذوي الإعاقة عادلة وضرورية.

.19تستحق أسر الأشخاص ذوي الإعاقة دعمًا اجتماعيًا إضافيًا.

.20ينبغي على المجتمع زيادة الاستثمار في الخدمات الداعمة لدمج الأشخاص ذوي الإعاقة.

القسم :8الأولويات المجتمعية لتمكين الأشخاص ذوي الإعاقة

.29ما المجال الذي ينبغي أن يحظى بالأولوية الوطنية القصوى لتعزيز دمج الأشخاص ذوي الإعاقة؟

التوعية المجتمعية والتعليم

تسهيل الوصول إلى خدمات الرعاية الصحية

الدمج في التعليم

فرص العمل

خدمات دعم الأسرة

جميعها مهمة بالقدر نفسه

.30ما الأساليب التي تعتقد أنها الأكثر فاعلية في تعزيز دمج الأشخاص ذوي الإعاقة؟)يمكن اختيار أكثر من إجابة(

حملات التوعية المجتمعية

التعليم المدرسي

تدريب العاملين في القطاع الصحي

إصلاح السياسات الحكومية

تحسين التمثيل الإعلامي للأشخاص ذوي الإعاقة

البرامج الأسرية والمجتمعية

نهاية الاستبيان

شكرًا لمشاركتك في هذه الدراسة .ستسهم إجاباتك في تعزيز الوعي المجتمعي بقضايا الإعاقة، ودعم الدمج الاجتماعي، وتطوير استراتيجيات تمكين الأشخاص ذوي الإعاقة في المملكة العربية السعودية.
